# Efficacy and safety of fixed-duration venetoclax plus obinutuzumab in untreated Japanese CLL and SLL: a phase 2 study

**DOI:** 10.1007/s12185-025-04095-w

**Published:** 2025-11-10

**Authors:** Koji Izutsu, Mitsumasa Watanabe, Tomomi Toubai, Taku Tsukamoto, Dai Maruyama, Takahiro Kumode, Noriko Fukuhara, Natsumi Ogawa, Natsuko Satomi, Yasuko Nishimura, Hideyuki Honda, Brenda Chyla, Jun Takizawa

**Affiliations:** 1 Department of Hematology, National Cancer Center Hospital, 5-1-1 Tsukiji, Chuo-ku, Tokyo, 104-0045 Japan; 2 Hyogo Prefectural Amagasaki General Medical Center, Hyogo, Japan; 3 Yamagata University Hospital, Yamagata, Japan; 4 University Hospital Kyoto Prefectural University of Medicine, Kyoto, Japan; 5 Cancer Institute Hospital, Japanese Foundation for Cancer Research, Tokyo, Japan; 6 Kindai University Hospital, Osaka, Japan; 7 Tohoku University Hospital, Miyagi, Japan; 8 AbbVie GK, Tokyo, Japan; 9 AbbVie Inc., North Chicago, IL USA; 10 Niigata University Faculty of Medicine, Niigata, Japan

**Keywords:** Chronic lymphocytic leukemia, Fixed-duration therapy, Obinutuzumab, Small lymphocytic lymphoma, Venetoclax

## Abstract

This phase 2 study (NCT05105841) evaluated the safety and efficacy of a fixed-duration 12-cycle regimen of venetoclax plus obinutuzumab in Japanese patients with previously untreated chronic lymphocytic leukemia (CLL) or small lymphocytic lymphoma (SLL). The primary efficacy endpoint was the complete remission (CR)/complete remission with incomplete marrow recovery (CRi) rate, assessed by an independent review committee (IRC) according to the 2008 International Workshop on CLL criteria. Ten patients (6 male, 4 female; 9 CLL, 1 SLL) with a median age of 69.5 years (range 52–76) received venetoclax for a median duration of 11.3 months (range 9.2–12.4). The IRC-assessed CR/CRi rate based on the best overall response was 90.0% (95% confidence interval 55.5%, 99.7%). All patients experienced at least one treatment-emergent adverse event (TEAE), and three patients (30.0%) experienced at least one serious TEAE. The most common TEAEs included infusion-related reactions (60.0%), decreased neutrophil count (50.0%), and nausea (40.0%). Nine patients (90.0%) experienced TEAEs related to venetoclax, while all ten patients (100.0%) had TEAEs related to obinutuzumab. One patient (10.0%) developed COVID-19 pneumonia, necessitating the discontinuation of venetoclax. These findings demonstrate the high efficacy and manageable safety profile of venetoclax plus obinutuzumab in this patient population.

**Supplementary Information:**

The online version contains supplementary material available at 10.1007/s12185-025-04095-w.

## Introduction

Chronic lymphocytic leukemia (CLL) is a B-cell malignancy characterized by lymphocytosis, leukemic infiltration of the bone marrow, lymphadenopathy, and splenomegaly [1]. CLL and small lymphocytic lymphoma (SLL) are considered manifestations of the same disease entity, with the distinction that patients with SLL typically present with lymphadenopathy and clonal B-cell infiltration of lymph nodes, exhibiting the same immunophenotype as CLL cells, but without an increase in clonal B cells in peripheral blood [1]. CLL is the most prevalent form of leukemia among adults in many European and North American populations, but is less common in Asian countries [2], suggesting a potential role of genetic predisposition in disease susceptibility. The incidence of CLL increases with age, peaking at around 75–80 years [2].

The clinical course of CLL is generally indolent, but exhibits considerable interpatient variability. Dysregulation of the apoptotic process is a hallmark of CLL pathogenesis, with B-cell lymphoma 2 (BCL-2) protein playing a critical regulatory role [3]. Venetoclax, an orally administered, highly selective BH3 mimetic, acts as a BCL-2 antagonist [4, 5]. In the MURANO study, which involved patients with relapsed or refractory CLL, a fixed-duration 2-year regimen of venetoclax plus rituximab demonstrated sustained survival benefits, including improved 5-year overall survival (OS), prolonged median progression-free survival (PFS), and extended minimal residual disease (MRD) doubling time compared with bendamustine plus rituximab [6]. Additionally, in the CLL14 study, involving treatment-naïve patients with coexisting conditions, 12 cycles of venetoclax combined with obinutuzumab, an anti-CD20 monoclonal antibody, resulted in a significantly longer median PFS compared with the chlorambucil plus obinutuzumab regimen, with a median follow-up of 76.4 months [7]. In the United States, treatment guidelines recommend venetoclax, in combination with either obinutuzumab or Bruton tyrosine kinase (BTK) inhibitors (e.g., ibrutinib, acalabrutinib), as a first-line treatment for patients with symptomatic or progressive CLL [8]. In Japan, however, venetoclax is currently approved only for the treatment of relapsed or refractory CLL/SLL and is not indicated for previously untreated cases. According to the latest guidelines issued by the Japanese Society of Hematology, first-line regimens prioritize BTK-based therapies, such as ibrutinib monotherapy and acalabrutinib with or without obinutuzumab, while venetoclax-based combination therapies are not listed among the preferred initial treatment options [9]. Given the advanced age at diagnosis and the high burden of comorbidities observed among Japanese patients with CLL/SLL [10], there remains a significant unmet need for well-tolerated, fixed-duration targeted therapies in this population.

In this phase 2 study, we evaluated the safety and efficacy of 12 fixed-duration cycles of venetoclax in combination with obinutuzumab in Japanese patients with previously untreated CLL/SLL. The primary efficacy endpoint was the rate of complete remission (CR) or complete remission with incomplete marrow recovery (CRi).

## Materials and methods

### Study design

This phase 2, open-label, multicenter, non-comparator, two-cohort study (NCT05105841) evaluated the safety and efficacy of treatment regimens in Japanese patients with previously untreated CLL or SLL. Patients were assigned to one of two treatment cohorts using a 1:1 variable alternating assignment methodology: Cohort 1 received venetoclax in combination with obinutuzumab, while Cohort 2 was administered venetoclax with ibrutinib, which is separately reported. The study comprised three distinct phases: screening, treatment, and follow-up.

The study adhered to the ethical principles outlined in the 1964 Declaration of Helsinki and its subsequent amendments, along with the International Conference on Harmonization good clinical practice guidelines. The study protocol, informed consent documents, recruitment materials, and all subject-facing materials received approval from the institutional review boards or ethics committees at each participating institution. Written informed consent was obtained from all patients prior to study enrollment.

### Patients

Eligible patients met specific inclusion criteria, including (i) age ≥ 65 years, or 20–64 years with either a total cumulative illness rating scale (CIRS) score [11] > 6 or a creatinine clearance < 70 mL/min as estimated by the Cockcroft–Gault equation; (ii) Eastern Cooperative Oncology Group performance status ≤ 2; (iii) measurable nodal disease, defined as at least one lymph node > 1.5 cm in the longest diameter on computed tomography imaging; (iv) active CLL/SLL requiring treatment according to the 2008 International Workshop on CLL (iwCLL) criteria [12]; and (v) no prior treatment for CLL/SLL.

### Treatment

**Fig. 1:**
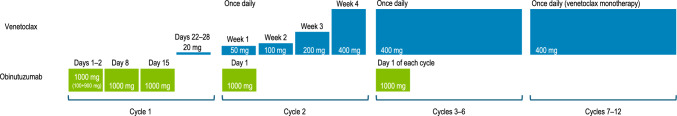
Treatment schedule. Obinutuzumab (1000 mg) was administered intravenously on day 1 (or 100 mg on day 1 and 900 mg on day 2), day 8, and day 15 of cycle 1, followed by 1000 mg on day 1 of cycles 2–6. Venetoclax was administered orally once daily on day 22 of cycle 1 with a weekly dose ramp-up schedule of 20, 50, 100, 200, and 400 mg

Patients were treated with obinutuzumab and venetoclax following the same regimen established in the CLL14 study [13]. During cycle 1, patients received obinutuzumab intravenously on day 1 at either 100 mg or 1000 mg (with an additional 900 mg administered on day 2 if the initial dose was 100 mg), followed by 1000 mg on days 8 and 15 (Fig. 1). For cycles 2 through 6, a consistent dose of 1000 mg of obinutuzumab was administered on day 1. Venetoclax was administered orally once daily, with a dose-escalation schedule of 20, 50, 100, 200, and 400 mg over 5 weeks, continuing at a maintenance dose of 400 mg once daily until the completion of cycle 12.

Premedication for infusion-related reaction prophylaxis consisted of oral acetaminophen and an antihistamine, administered 30–60 min prior to each obinutuzumab infusion. Additionally, on days 1 and 2 of cycle 1, patients received an intravenous corticosteroid (100 mg prednisolone or equivalent) at least 1 h prior to obinutuzumab administration.

### Endpoints and assessments

The primary efficacy endpoint was the CR/CRi rate, as assessed by an independent review committee (IRC) according to the 2008 iwCLL. The CR/CRi rate was defined as the proportion of patients whose best response achieved CR or CRi. Efficacy assessments were conducted on day 1 of cycles 4, 7, and 9, 30 days following the end of treatment (EOT), and every 3 months thereafter. Secondary efficacy endpoints included OS, the CR/CRi rate as assessed by an investigator according to the 2008 iwCLL criteria, and additional outcomes such as overall response rate (ORR), PFS, duration of response (DOR), and time to progression (TTP), assessed by both the investigator and the IRC according to the 2008 iwCLL criteria. ORR was defined as the proportion of patients who achieved a best overall response of CR, CRi, nodular partial remission (nPR), or partial remission (PR).

Safety and tolerability were evaluated throughout the study, including the monitoring of treatment-emergent adverse events (TEAEs), physical examinations, vital sign assessments, and laboratory testing. Adverse events (AEs) of particular interest in Cohort 1 included laboratory and clinical tumor lysis syndrome (TLS) as per the Howard Criteria [14] and infusion-related reactions (IRRs) occurring during infusion or within 24 h after the end of infusion. TLS risk was determined at baseline and prior to the initiation of venetoclax therapy. Details regarding TLS risk assessment, prophylactic interventions, and management of electrolyte imbalances have been previously detailed [15]. Adverse events were categorized using the Medical Dictionary for Regulatory Activities (MedDRA) version 26.1, with severity graded according to the National Cancer Institute Common Terminology Criteria for Adverse Events version 4.03. To assess the initial safety and tolerability of these regimens in the Japanese population, dose-limiting toxicities (DLTs) were monitored in the first six patients starting from cycle 1, day 22 of venetoclax administration for a minimum of five weeks, including at least one week of venetoclax dosing at 400 mg (Supplementary Information). Enrollment of additional patients proceeded only if ≤ 1 DLT was observed within this initial cohort.

Exploratory endpoints included the undetectable measurable residual disease (uMRD) rate at 3 months post-EOT, defined as the proportion of patients achieving uMRD at this time point, and the overall uMRD rate, representing the proportion of patients with uMRD on day 1 of cycle 9, 3 months post-EOT, or both. uMRD was defined as fewer than one CLL cell per 10,000 leukocytes (10^–4^) in peripheral blood, as determined by flow cytometry.

### Statistical analysis

The full analysis set (FAS) included all patients who received at least one dose of venetoclax, obinutuzumab, or both agents. The per-protocol (PP) population comprised patients within the FAS who had evaluable disease at baseline. To establish a CR/CRi rate of ≥ 10% (predefined efficacy threshold) with a statistical power of ≥ 80% and a two-tailed significance level of 0.05, a minimum of 10 patients per cohort in the PP population was required. The treatment regimen was deemed effective if the lower boundary of the 95% confidence interval (CI) for the observed CR/CRi rate exceeded 10%.

Efficacy assessments of primary and secondary endpoints conducted by an IRC were analyzed in the PP population, while those assessed by investigators were analyzed in the FAS. Point estimates and corresponding 95% Clopper–Pearson exact CIs were calculated for CR/CRi and ORR. Time-to-event endpoints, including PFS, DOR, OS, and TTP, were evaluated using the Kaplan–Meier method. Safety analyses were performed using the FAS.

## Results

### Patients and treatment

The study was conducted at 11 sites across Japan, enrolling 10 patients (6 males, 4 females), with a median age of 69.5 years (range 52–76). Among the participants, 9 patients were diagnosed with CLL and 1 with SLL (Table 1). TLS risk was categorized as high in 2 patients (20.0%) and medium in 5 patients (50.0%). Comorbidity assessment revealed that 5 patients (50.0%) had a CIRS score greater than 6, and 6 patients (60.0%) exhibited a creatinine clearance of < 70 mL/min. The median duration of study participation was 19.5 months (range 10.2–26.7) as of the data cutoff on April 11, 2024. All patients received 6 cycles of obinutuzumab, and the median duration of venetoclax treatment was 11.3 months (range 9.2–12.4).

**Table 1:** Patient characteristics (FAS)

Characteristics	Venetoclax + Obinutuzumab N = 10
Age, years	
Median (range)	69.5 (52–76)
≥ 65, n (%)	6 (60.0)
≥ 75, n (%)	2 (20.0)
Sex, n (%)	
Male	6 (60.0)
Female	4 (40.0)
Diagnosis and stage	
CLL, n (%)	9 (90.0)
Binet stage, n (%)^a^	
A	3 (33.3)
B	4 (44.4)
C	2 (22.2)
SLL, n (%)	1 (10.0)
Lugano classification^b^, n (%)^c^	
IV	1 (100.0)
17p deletion, n (%)	
Present	1 (10.0)
Absent	9 (90.0)
ECOG performance status, n (%)	
0	10 (100.0)
TLS risk category, n (%)	
High	2 (20.0)
Medium	5 (50.0)
Low	3 (30.0)
Total CIRS score	
Median (range)	5.5 (0–12)
> 6, n (%)	5 (50.0)
Tumor bulk^d^, cm	
< 5, n (%)	9 (90.0)
≥ 5 and < 10, n (%)	1 (10.0)
Creatinine clearance, mL/min	
Median (range)	64.755 (34.85–94.81)
< 70, n (%)	6 (60.0)

CIRS, cumulative illness rating scale; CLL, chronic lymphocytic leukemia; ECOG, Eastern Cooperative Oncology Group; FAS, full analysis set; SLL, small lymphocytic lymphoma; TLS, tumor lysis syndrome

^a^Proportion among patients with CLL

^b^Modified version of the Ann Arbor staging system

^c^Proportion among patients with SLL

^d^Calculated using the largest diameter among measurable lesions

### Efficacy

**Fig. 2:**
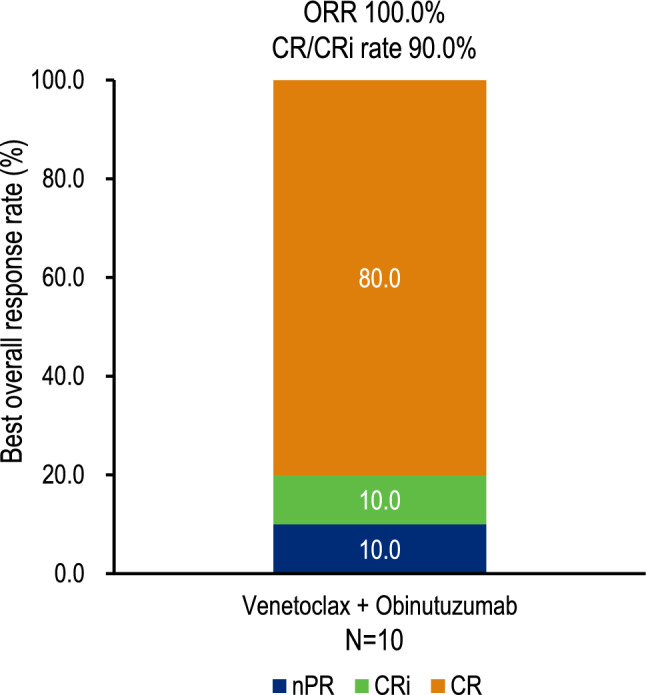
Best overall response assessed by an IRC according to the 2008 iwCLL criteria. The CR/CRi rate was 90.0%, and the ORR was 100.0%. Among the patients, 80.0% achieved CR, 10.0% achieved CRi, and 10.0% achieved nPR. CR, complete remission; CRi, complete remission with incomplete marrow recovery; IRC, independent review committee; iwCLL, International Workshop on Chronic Lymphocytic Leukemia; nPR, nodular partial remission; ORR, overall response rate

**Fig. 3:**
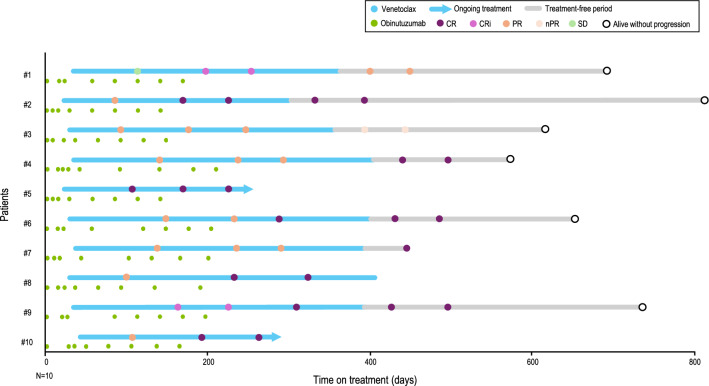
Overall response and time on treatment for each patient. A swimmer plot shows the duration of treatment, the IRC-assessed overall response until the visit 3 months after the last study treatment (no earlier than 2 months after the end of study treatment), and the last observation for each patient. CR, complete remission; CRi, complete remission with incomplete marrow recovery; IRC, independent review committee; nPR, nodular partial remission; PR, partial response; SD, stable disease

The CR/CRi rate based on the best overall response per patient was 90.0% (95% CI: 55.5%, 99.7%), with the lower limit of the CI exceeding the pre-specified efficacy threshold of 10% (Fig. 2). The ORR, as assessed by an IRC, was 100.0% (95% CI: 69.2%, 100.0%). Of the patients, 80.0% achieved CR, 10.0% achieved CRi, and 10.0% achieved nPR. Median DOR, PFS, OS, and TTP were not estimated, as no patients experienced disease progression or death during the study. The overall response at each disease assessment visit and duration of treatment is summarized in the swimmer plot (Fig. 3), with 8 patients on study treatment completion. The overall uMRD rate was 100.0% (10/10) in the full analysis set with all patients achieving uMRD at cycle 9, day 1. At 3 months post-EOT, the uMRD rate was 60.0% (6/10) overall and 100.0% (6/6) among the evaluable patients. The remaining 4 patients were not evaluated because they had not reached the 3-month post-EOT visit at the time of the data cutoff.

### Safety

All 10 patients experienced at least one TEAE during the study (Table 2). Grade 3 or 4 TEAEs were observed in 8 patients (80.0%). Venetoclax-related TEAEs occurred in 9 patients (90.0%), while all patients (100.0%) had at least one TEAE associated with obinutuzumab. Serious TEAEs were reported in 3 patients (30.0%), including macular hole (n = 1), ureterolithiasis (n = 1), and febrile neutropenia (n = 1). The serious febrile neutropenia on day 4 of cycle 1 was possibly related to obinutuzumab before venetoclax initial dose. The serious macular hole and ureterolithiasis were not related to obinutuzumab or venetoclax. No deaths or DLTs occurred during the study. Treatment discontinuation, interruption, and dose reduction of venetoclax as a result of TEAEs were reported in 1 (10.0%), 6 (60.0%), and 7 (70.0%) patients, respectively. Discontinuation of venetoclax was necessitated by the development of COVID-19 pneumonia in a single patient.

**Table 2:** Treatment-emergent adverse events (≥ 3 patients in any grade, FAS)

Adverse events, n (%)	Venetoclax + Obinutuzumab N = 10	
	Any grade	Grade 3 or 4
Patients with any event	10 (100.0)	8 (80.0)
Patients with neutropenia or neutrophil count decreased	8 (80.0)	8 (80.0)
Blood and lymphatic system disorders		
Anemia	3 (30.0)	1 (10.0)
Leukopenia	3 (30.0)	3 (30.0)
Lymphopenia	3 (30.0)	3 (30.0)
Neutropenia^a^	3 (30.0)	3 (30.0)
Gastrointestinal disorders		
Constipation	3 (30.0)	0 (0.0)
Diarrhea	3 (30.0)	0 (0.0)
Nausea	4 (40.0)	0 (0.0)
General disorders and administration site conditions		
Pyrexia	3 (30.0)	0 (0.0)
Infections and infestations		
COVID-19	3 (30.0)	0 (0.0)
Injury, poisoning and procedural complications		
Infusion related reaction	6 (60.0)	1 (10.0)
Investigations		
Neutrophil count decreased	5 (50.0)	5 (50.0)
Platelet count decreased	3 (30.0)	3 (30.0)

Adverse events were classified by System Organ Class and coded using Preferred Terms according to MedDRA version 26.1

FAS, full analysis set; MedDRA, Medical Dictionary for Regulatory Activities

^a^Including a single case of febrile neutropenia

The most frequently reported TEAEs included infusion-related reactions (n = 6, 60.0%), decreased neutrophil count (n = 5, 50.0%), and nausea (n = 4, 40.0%). The most commonly observed Grade 3 or 4 TEAEs were decreased neutrophil count (n = 5, 50.0%), leukopenia, lymphopenia, neutropenia, and decreased platelet count (n = 3, 30.0% each) (Table 2). TEAEs leading to venetoclax dose interruption included COVID-19 (30.0%) and macular hole, aphthous ulcer, pneumonia, tracheitis, increased blood alkaline phosphatase, increased gamma-glutamyltransferase, and decreased neutrophil count (10.0% each). TEAEs resulting in venetoclax dose reduction included decreased neutrophil count (50.0%), neutropenia and decreased platelet count (20.0% each), and fungal pneumonia (10.0%). TEAEs leading to obinutuzumab interruption occurred in 8 patients (80.0%) and included decreased neutrophil count (50.0%), neutropenia (30.0%), and febrile neutropenia, leukopenia, thrombocytopenia, COVID-19, infusion-related reaction, injection-related reaction, increased aspartate aminotransferase, and decreased platelet count (10.0% each). A total of 8 patients (80.0%) experienced IRRs related events during or within 24 h of obinutuzumab infusion, with a median onset on day 1 (range 1–3 days) following infusion initiation and a median duration of 2 days (range 1–6 days). The preferred terms recorded in MedDRA for these IRRs included infusion-related reaction (n = 6, 60.0%), pyrexia (n = 2, 20.0%), and injection-related reaction (n = 1, 10.0%). One patient experienced a Grade ≥ 3 infusion-related reaction, all of which were non-serious. No TLS events were observed.

## Discussion

In Cohort 1 of this phase 2 study, a fixed-duration regimen of 12 cycles of venetoclax plus obinutuzumab demonstrated robust efficacy and an acceptable safety profile in 10 Japanese patients with previously untreated CLL/SLL. The CR/CRi rate based on the best overall response and assessed by an IRC according to the 2008 iwCLL criteria was 90.0% (95% CI 55.5%, 99.7%), thereby exceeding the predefined efficacy threshold of 10% for the primary endpoint.

Secondary efficacy outcomes further supported the therapeutic potential of this regimen. The ORR, also assessed by the IRC, was 100.0% (95% CI 69.2%, 100.0%). Both the overall uMRD rate during the study and the uMRD rate at 3 months post-EOT among evaluable patients were 100.0%, indicating rapid and profound disease remission. These findings are largely consistent with results from the global phase 3 CLL14 trial, which demonstrated superior outcomes for venetoclax plus obinutuzumab in previously untreated patients with CLL and coexisting conditions [13].

Regarding safety, in the initial report of the CLL14 study with a median follow-up of 28.1 months, AEs of any grade were reported in 94.3% of patients treated with venetoclax plus obinutuzumab, with Grade 3 or 4 neutropenia occurring in 52.8% [13]. The all-cause mortality rates were 9.3% for the venetoclax plus obinutuzumab group and 7.9% for the chlorambucil plus obinutuzumab group [13]. In contrast, in the present study, all 10 patients experienced at least one TEAE, and one patient (10%) developed serious febrile neutropenia possibly related to obinutuzumab. Notably, no deaths or DLTs were reported, suggesting that the regimen was well tolerated in this patient cohort.

While the CLL14 study targeted treatment-naïve patients with coexisting conditions [13], the recent global phase 3 GAIA/CLL13 study demonstrated the favorable efficacy and safety of venetoclax plus obinutuzumab compared with both chemoimmunotherapy and venetoclax–rituximab in previously untreated fit patients with CLL [16]. The findings from these studies, together with those of the present investigation, support the clinical benefit of this regimen across both fit and unfit patient populations.

However, several limitations of the current study warrant consideration. The non-randomized, open-label design precludes direct statistical comparisons with standard regimens recommended in Japan. Additionally, the small sample size (N = 10), which is primarily due to the low incidence of CLL/SLL in Japan, limits the generalizability of the findings. Furthermore, the absence of disease progression or deaths during the study may be attributable to the relatively short follow-up period. Further studies with larger patient populations and extended follow-up are warranted to confirm the long-term survival benefit and safety profile of venetoclax plus obinutuzumab.

In conclusion, the present study provides evidence of the high efficacy and manageable safety profile of fixed-duration venetoclax plus obinutuzumab as a first-line treatment for Japanese patients with previously untreated CLL/SLL.

## Supplementary Information

Below is the link to the electronic supplementary material.

Supplementary file1 (DOCX 32 KB)

## Declarations

### Data availability

AbbVie is committed to responsible data sharing regarding the clinical trials we sponsor. This includes access to anonymized, individual, and trial-level data (analysis data sets), as well as other information (e.g., protocols, clinical study reports, or analysis plans), as long as the trials are not part of an ongoing or planned regulatory submission. This includes requests for clinical trial data for unlicensed products and indications. These clinical trial data can be requested by any qualified researchers who engage in rigorous, independent, scientific research, and will be provided following review and approval of a research proposal, Statistical Analysis Plan (SAP), and execution of a Data Sharing Agreement (DSA). Data requests can be submitted at any time after approval in the US and Europe and after acceptance of this manuscript for publication. The data will be accessible for 12 months, with possible extensions considered. For more information on the process or to submit a request, visit the following link: https://vivli.org/ourmember/abbvie/ then select “Home”.
