# Supplementary material for: Efficacy and safety of fixed-duration venetoclax plus obinutuzumab in untreated Japanese CLL and SLL: a phase 2 study
Source: Int J Hematol. 2025 Nov 10;123(2):225–32. doi: 10.1007/s12185-025-04095-w (PMC12913321; doi:10.1007/s12185-025-04095-w)
Supplement: Supplementary file 1 — Supplementary file1 (DOCX 32 KB) [file 12185_2025_4095_MOESM1_ESM.docx]

Supplementary information

Efficacy and safety of fixed-duration venetoclax plus obinutuzumab in untreated Japanese CLL and SLL: a phase 2 study

Koji Izutsu et al.

**Supplementary methods: Dose-limiting toxicity (DLT) assessment**

**1.** **DLT assessment**

To evaluate the initial safety and tolerability of venetoclax in combination with obinutuzumab in Japanese patients, DLTs were assessed in the first six patients enrolled in the study. The DLT assessment period commenced on cycle 1 day 22 and continued for a minimum duration of 5 weeks, which included at least 1 week of venetoclax administration at the target dose of 400 mg. Enrollment of subsequent patients was permitted only if no more than 1 DLT occurred within this initial cohort. Patients who failed to receive ≥80% of the planned doses of venetoclax or obinutuzumab for reasons unrelated to toxicity (e.g., non-compliance, withdrawal of consent, disease progression) or who did not complete the DLT assessment period for any reason other than DLTs were considered non-evaluable. In such cases, replacement patients were enrolled to ensure that six evaluable patients were assessed for DLTs.

**2. Definition of DLTs**

Adverse events (AEs) meeting any of the following criteria were defined as DLTs if deemed reasonably related to the administration of venetoclax and/or obinutuzumab and not attributable to other identifiable causes (e.g., tumor progression, concurrent illness, or concomitant medication), as determined by the investigator.

1. Grade 4 neutropenia that was absent at screening, unresponsive to granulocyte colony-stimulating factors, and persisted for >14 days
2. Grade 3 or 4 febrile neutropenia with fever lasting >4 days
3. Grade 4 thrombocytopenia associated with bleeding or thrombocytopenia not recovering to Grade ≤2 or ≥80% of baseline platelet count (whichever was lower) within 4 weeks
4. Clinical tumor lysis syndrome (TLS) as defined by the Howard criteria
5. Grade 4 infusion-related reaction occurring during or within 24 hours following obinutuzumab infusion despite appropriate premedication and infusion rate adjustment. Grade 3 infusion-related reactions to obinutuzumab that resolved with treatment and did not require >24-hour dose delay were not classified as DLTs.
6. Grades 3, 4, or 5 AEs persisting for >2 weeks with or without treatment, excluding the following:

• Grade 3 neutropenia without fever not resolved within 4 weeks

• Grade 3 thrombocytopenia without bleeding not resolved within 4 weeks

• Grades 3 or 4 lymphopenia and/or leukopenia

• Grade 3 anemia associated with extensive bone marrow infiltration by chronic lymphocytic leukemia cells not resolved within 4 weeks

• Grade 3 laboratory TLS resolved within 72 hours

• Grade 3 or 4 hyperuricemia or hypocalcemia or Grade 3 hyperkalemia resolved within 72 hours without clinical signs of TLS

• Grade 3 hyperphosphatemia requiring hospitalization solely for monitoring or prophylaxis

• Grade 3 or 4 elevations in liver function tests (e.g., aspartate transaminase, alanine transaminase, alkaline phosphatase) attributable to obinutuzumab and resolving to Grade 2 within 7 days without clinical symptoms

• Grade 3 or 4 bilirubin elevation due to hemolysis resolved within 14 days

• Grade 3 nausea, vomiting, and/or diarrhea that responded to treatment
